# A Review of Pathophysiology, Molecular Mechanisms, and Omics Approaches of Spinal Cord Injury

**DOI:** 10.3390/ijms26167895

**Published:** 2025-08-15

**Authors:** Milan Patel, Alison J. Deng, Jamal Hasoon, Sayed Wahezi, Alaa Abd-Elsayed

**Affiliations:** 1 Department of Anesthesiology, University of Wisconsin School of Medicine and Public Health, Madison, WI 53726, USA; mpatel0503@gmail.com; 2 Department of Medicine, Washington University School of Medicine in St. Louis, St. Louis, MO 63110, USA; a.j.deng@wustl.edu; 3 Department of Anesthesiology, Critical Care, and Pain Medicine, The University of Texas Health Science Center at Houston, Houston, TX 77030, USA; jamal.j.hasoon@uth.tmc.edu; 4 Departments of Rehabilitation Medicine, Anesthesiology, and Orthopedic Surgery, Montefiore Medical Center, Bronx, NY 10461, USA; swahezi@montefiore.org

**Keywords:** spinal cord injury (SCI), pathophysiology, molecular mechanisms, omics, genomics, proteomics, metabolomics, neuroinflammation, mitochondrial dysfunction, biomarkers

## Abstract

Spinal cord injuries are often devastating and result in severe functional limitations. Our review breaks down the pathophysiology, molecular mechanisms, and omics approaches regarding spinal cord injuries. The pathophysiology can be divided into two main phases, with the secondary phase being of greater interest. Understanding the underlying mechanisms behind these phases allows for targeted approaches to be developed. Advancements in omics technologies (genomics, epigenomics, transcriptomics, proteomics, and metabolomics) are excellent tools in creating tailored spinal cord injury treatment plans. Emerging therapeutic solutions involving ion imbalance, oxidative stress, and mitochondrial dysfunction also show promising results. Mitochondrial transplantation has shown promising initial results in maintaining cellular homeostasis and reducing inflammation. However, significant challenges remain in translating the omics and therapeutic approaches from animal models to clinical trials.

## 1. Introduction

Spinal cord injuries (SCIs) can be traumatic, degenerative, rheumatological, or neoplastic in origin. The purpose of this manuscript is to discuss traumatic SCI. Depending upon the level of injury, SCI can be debilitating and cause paralysis, pain, pressure sores, weakness, loss of bladder and bowel control, or difficulty breathing. The early diagnosis and treatment of SCI and its secondary conditions are critical to improving quality of life and increasing life expectancy. However, the treatments currently available are limited [1,2].

Millions globally live with SCIs, and that number is growing. The World Health Organization estimated that in 2021, 15.4 million people suffered with some type of SCI [3]. Another estimate in 2019 found that 20.6 million people worldwide were living with SCI [4]. The same study found an incidence rate of 0.9 million new cases and a global burden of years living with disability of 6.2 million years [4]. From statistics gathered by the National Spinal Cord Injury Statistical Center, the average age at injury is 42 years; males represent about 80% of new SCIs [5]. Men are more likely to participate in risky behavior, such as reckless driving, extreme sports, and riding motorcycles, or work physically demanding jobs, which in turn are all more likely to lead to SCI [6,7]. There are some investigations into the biological factors behind the gender difference in SCI incidence, including the protective potential of female hormones estrogen and progesterone found in mice [8,9]. Due to the increased incidence and burden of disease of SCIs [3,4], the need to understand and treat SCIs effectively is of paramount importance.

Studying the pathophysiology and molecular medicine of SCIs is crucial for developing effective therapies as it serves as a basis for identifying treatment targets. More therapeutic avenues may arise from the solid foundational knowledge base for the pathophysiology of SCIs to enhance the breadth of therapies currently available. More emerging therapies may arise from further elucidating the complex pathways that underlie SCI and its presentation [10,11].

More recently, omics approaches, which include genomics, transcriptomics, proteomics, and metabolomics, provide promising potential in identifying therapeutic targets. These omics approaches are particularly advantageous in mapping the spatial organization of cells and the interactions between cells, creating a microenvironment [12]. These unique and distinct microenvironments can influence cell fate, phenotype, and function. Spatial multi-omics show promise in contextualizing a heterogeneous microenvironment after an SCI that can lead to precision medicine treatments [13]. Therefore, practitioners and researchers must study and understand omics methods further.

Before exploring deeper into the contents of our review. Information regarding the selection and search parameters for articles used in this review is important to discuss as well. The authors mainly used the database of PubMed in order to find the most current and relevant studies. The initial outline and subheadings guided the search terms entered, and thus inclusion of specific studies was conducted based on overall relevance and significance to the overall manuscript.

## 2. Pathophysiology of Traumatic Spinal Cord Injury

SCIs are often caused by traumatic events, most commonly with vehicle crashes (38%), followed by falls (30.5%), acts of violence (13.5%), and sports (9%) [14]. There are two stages of SCI: primary injury and secondary injury [15].

### 2.1. Primary Injury Phase

In most cases, mechanical trauma is responsible for initiating the primary stage of SCIs. Trauma is inflicted by a physical force, commonly compression, shearing, laceration, or stretching. This traumatic event results in the damage of axons, hemorrhage, and disruption of vasculature and neural membranes [11,15]. The severity of the SCI depends on the location, duration, and extent of the initial event [16].

### 2.2. Secondary Injury Phase

Biochemical, mechanical, and physiological changes characterize the secondary injury phase. These changes lead to degeneration in the spinal cord and are responsible for the majority of post-traumatic neurodegeneration [17,18]. The secondary phase is divided into three phases: acute, sub-acute, and chronic. Glial cell death and ischemia in the primary phase trigger the immune system cascade [19]. The aim of the recruited cells is to repair damage and restore homeostasis [20,21].

Excitotoxicity, or overstimulation, in neurons is caused by a high concentration of glutamate released following SCI. An abundant amount of glutamate triggers overactivation of glutamate receptors, and a subsequent imbalance of ions leads to neuronal and glial death [16]. Prolonged overactivation of glutamate receptors is damaging and will cause necrosis and apoptosis in the damaged area [22,23].

Additionally, oxidative stress is known to have an essential role in secondary injury [18]. The oxygen radical cascade is in response to elevated intracellular Ca^2+^. The reduction of an oxygen molecule (O_2_) produces a superoxide radical (O_2_^•−^) that can react with other molecules either as an oxidant or reductant to generate potentially more reactive oxygen species (ROS). Specifically, superoxide is a reducing agent in the spontaneous dismutation reaction to hydrogen peroxide: 2O_2_^•−^ + 2H^+^ → H_2_O_2_ + O_2_. Lipid peroxidation is the predominant form of ROS-induced damage, triggered by an oxidative attack on cell membrane polyunsaturated fatty acids [18,24]. Ultimately, this process generates lipid radicals and damages neuronal cells and mitochondrial membranes. This damage to the mitochondrial membrane alongside oxidation of proteins and mitochondrial DNA (mtDNA) may lead to mitochondrial dysfunction [25]. mtDNA mutations and the loss of function of respiratory chain proteins in the mitochondria affect metabolism and cellular respiration, key functions of the organelle [26,27]. Mitochondrial dysfunction can cause limb function impairments and neurological deficits, including paralysis [28].

Excitotoxins, ROS, and inflammatory mediators can initiate cell necrosis, cell death caused by injury, or apoptosis, i.e., programmed cell death [29]. Secondary injury after an SCI is due to apoptosis of neurons and oligodendrocytes in the spinal cord. Without these critical neurological cells, there are long-term neurological deficits. Neurons and glial cells were both first observed at four hours post-SCI. Neuronal death peaks at eight hours following the SCI incident. Glial cell apoptosis peaks at 24 h [29]. Oligodendrocyte and microglia apoptosis is highest eight days after SCI and is found in the tissue surrounding the injury site [29]. These cells are first observed at 24 h post-SCI. The eight days to peak apoptosis timeline is due to the time necessary for secondary-phase molecular mechanisms including inflammation, excitotoxicity, and oxidative stress [30,31,32]. This delay between injury and apoptosis provides an excellent opportunity for therapeutic interventions to mitigate ongoing and potential damage. It was found that apoptosis contributes to tissue damage, as seen after SCI [29]. The caspase-independent pathway regulates necrosis and has an emerging role in cell death and tissue damage post-SCI [29].

In the chronic phase, beginning six months after trauma, glial scar formation matures and syrinxes develop. The scar contains the inflammation at the injured site. The scar has cellular and molecular components that act as a physical and chemical barrier that prevents axon regeneration [33]. Without proper axon regeneration, the injury site is permanently damaged and can cause long-lasting adverse effects, including physical and neurological impairments [34].

## 3. Molecular Mechanisms Underlying SCI

Microglia, macrophages, neutrophils, and pro-inflammatory cytokines are recruited to the injury site during the secondary injury phase [11,19,35]. Microglia, the main macrophages in the central nervous system, participate in phagocytosis to clear the injury site and secrete pro-inflammatory cytokines. Pro-inflammatory cytokines include tumor necrosis factor alpha (TNF-α), interleukin-1β (IL-1β), and interleukin-6 (IL-6), which signal to mediate inflammation and recruit immune cells to the injury site as part of the inflammatory mechanisms [19,36]. Neutrophils and macrophages infiltrate the injury site, begin secreting cytokines, and clear debris by phagocytosis [16]. While initial inflammation benefits repair, chronic inflammation can lead to additional cell death, spinal cord damage, and pain [37]. Additionally, the overwhelming response from neutrophils, macrophages, and microglia can prevent repair and tissue damage [19].

There has been an interest in specifically in interleukin-10 (IL-10). In addition to the previously mentioned interleukins, IL-10 has also been found to aid in regulating inflammation, oxidative stress, neuronal apoptosis, and glial scars post-SCI [38]. Animal studies have analyzed these properties; however, their neuroprotective effects should be further studied in humans, with the previous literature suggesting their potential benefits.

Glutamate excitotoxicity is a mechanism by which excessive activation of glutamate receptors results in neuronal cell death [17]. Membrane damage from initial injury can activate calcium (Ca^2+^) channels, triggering the release of the neurotransmitter glutamate. Glutamate is an excitatory neurotransmitter that binds to ionotropic receptors, most notably N-methyl-D-aspartate (NMDA) receptors. These ligand-gated NMDA receptors require glutamate and glycine and postsynaptic membrane depolarization to open [39]. Once opened, NMDA receptors will allow further Ca^2+^ influx. If a large, unregulated amount of calcium enters the cell, it leads to cell death [39]. NMDA receptors are involved in the acute and chronic SCI excitotoxicity cascades. While there is some interest in NMDA-targeting drugs, such as the antagonist ketamine in treating SCI, evidence is limited to treating pain or SCI animal studies [40,41].

Ion dysregulation after an SCI can lead to increased protons into mitochondria, which reduces the membrane potential of the electron transport chain and results in a loss of ATP synthesis. This primary bioenergetic compound sustains cellular function. In addition, water flows into the mitochondrial matrix and causes swelling, eventually leading the mitochondria to rupture [42].

ROS can cause irreversible damage to DNA, lipids, and proteins, which prevents cellular function and eventually leads to cell death [43]. ROS-induced DNA oxidation can cause DNA breaks or mutations. For cell membranes, ROS oxidation of an unsaturated lipid leads to the formation of a hydroperoxidized lipid and an alkyl radical. As a result, it alters the lipid structure, affecting the membrane fluidity [44]. Finally, ROS can damage the structure of proteins and enzymes, causing a loss of function [44]. DNA, lipids, and protein damage are particularly impactful in mitochondria [25]. Mitochondria are essential to cellular function and the primary bioenergetic organelle. The impact of oxidative stress and ROS on mitochondria can lead to cell death [44].

At the core of the lesion are fibroblasts, which are part of connective tissue and produce fibrotic elements, as well as immune cells [33]. The lesion border, the penumbra, primarily comprises astrocytes, a glial cell that protects neurons. Type A pericytes surround the lesion core and proliferate in response to injury, contributing to scar formation [45]. This fibrotic scar hinders axonal regeneration as it is a physical barrier to axon generation and development [33]. The scar is also a chemical barrier: immune cells and overexpressed extracellular matrix components inhibit axon growth [33,46]. Understanding these molecular mechanisms provide an opportunity to develop targeted therapies.

## 4. Role of Omics Approaches in SCI Research

We can more efficiently identify targets and screen potential therapeutic drugs with knowledge of the molecular mechanisms of SCI damage. Omics are the fields that measure biological models [47]. In this review, we will focus on genomics, proteomics, and metabolomics, providing a broad overview of techniques, including current work, and their role in driving SCI research in the future.

Genomic studies provide a map of an individual’s genome. From many techniques, genomic analyses can capture single-nucleotide polymorphisms, insertions and deletions, and copy number variations [47]. These techniques include Sanger sequencing, next-generation sequencing, fluorescent in situ hybridization (FISH), and microarray-based comparative genomic hybridization (aCGH) [48]. Sanger and next-generation sequencing provide nucleotide sequences from DNA; FISH highlights structural abnormalities on chromosomes; and aCGH detects copy number variations. Genomics is powerful as it can detect and provide personalized data for individuals. Genome-wide association studies (GWAS), for example, analyzed genomic data on a population level and identified genetic variants associated with human diseases [49]. Thus, GWAS studies identify genetic markers that are related to the risk of diseases. Genomics is further stratified into epigenomics and transcriptomics to characterize gene expression.

### 4.1. Genomics

#### 4.1.1. Epigenomics

Epigenetics characterizes reversible changes in gene expression without changes in the DNA sequence. Commonly, DNA methylation is a marker of gene silencing, and histone acetylation is a marker of gene activation. Epigenomics helps identify DNA modifications, including methylation or histone acetylation [49,50]. Metabolic disturbances especially from spinal cord injuries can lead to effects felt by chromatin-modifying enzymes such as DNA methyltransferases and histone deacetylases. This in turn affects DNA methylation and histone modifications due to metabolic pathway disruption. Chromatin Immunoprecipitation (ChIP) is an epigenomic method to identify DNA binding sites [48]. The availability of a DNA binding site can indicate whether it can be transcribed. If the binding site is blocked, transcription factors cannot bind, and the encoded protein will not be made [51,52].

These techniques are beneficial in detecting gene expression changes after SCI. Epigenetic modifications, such as axon regeneration, are associated with critical repair processes following SCI. For example, histone H4 protein acetylation regulates regeneration-associated gene expression [53]. Post-SCI, the H4 is hypoacetylated, leading to transcriptional repression. Therefore, understanding these changes can assist in developing treatments that target this upstream inhibition of SCI repair processes, such as targeting H4 hypoacetylation [53].

#### 4.1.2. Transcriptomics

Transcriptomics studies the intermediate RNA transcribed, reflecting the functional read-out aspects of DNA that produce protein. Transcriptomics analyses can identify which transcripts, including splice sites, and how much transcript is present [49]. The key techniques are Northern blot, qPCR, cDNA microarray, and RNA-seq [48].

These principles can assist in uncovering gene expression changes following SCI. An RNA sequencing study sought to observe changes in the contused spinal cord of rats at five different time points. The time points chosen were 1 h, 1 day, 1 week, 1 month, and 3 months post-SCI. The overall results indicated 14,257 genes were commonly expressed among all time points observed. Furthermore, 2841 differentially expressed genes (DEGs) were found to be expressed at any of the five time points [54]. In addition to the previously mentioned information on inflammatory markers, interleukin signaling and neutrophil degradation were also found [54]. Additionally, snRNA-seq can be used comprehensively to understand the profile of cell populations seen in the lumbar region of rodent populations after SCI [55]. These studies provide an excellent avenue for further research regarding the impact of genomic studies specifically for SCI patients. Treatment plans can be tailor-made to the individual by utilizing rodent models given the gene expression observed. Gene expression changes following an SCI can be critical to developing a treatment plan and identifying the best action plan.

### 4.2. Proteomics

Proteomics studies are conducted on proteins further to understand protein activity, pathways, and interactions. Proteomics techniques include enzyme-linked immunosorbent assay (ELISA), two-dimensional gel electrophoresis (2-DE), mass spectrometry (MS), and two-hybrid system screening [48]. ELISA can detect and quantify proteins; 2-DE detects proteins by separating proteins by isoelectric point and molecular mass; MS determines the ion mass-to-charge ratio; and two-hybrid screening is often used to detect protein interactions [48].

Proteomics were used to identify that endoplasmic reticulum protein 29 (ERp29) regulated genes associated with cell survival and apoptosis [53]. ERp29 improved locomotor function in a mouse model by enhancing neuronal survival and axonal regeneration [56].

With an emphasis on acute SCI in this study, proteomics has been linked to application in further creating tailored post-SCI treatment plans. Using cerebrospinal fluid (CSF) and serum samples from 111 acute SCI patients, Skinnider et al. also ran a parallel project using the same proteomic methods on a pig model. In a human study, 21 healthy individuals were selected to serve as a negative control. As a result of the CSF and serum samples collected over the course of this study, proteomic portraits could be created in which variations in protein composition of acute SCI individuals could be tracked across the duration of the study. The results in the acute SCI individuals showed significant change, with approximately 39% of targeted proteins displaying substantial differences compared to the control individuals over the first 24 h. This study highlights the burgeoning significance of proteomics in further identifying SCI treatment plans [57].

### 4.3. Metabolomics

Metabolomics applies to amino acids, fatty acids, carbohydrates, and other cellular metabolic products [49]. In analyses, metabolomics quantifies the levels of these metabolites as a representation of metabolic function compared to normal values. Metabolite levels out of the normal range may indicate disease [49].

Specifically in SCI, metabolomics profiling can help locate potential biomarkers indicating SCI. A practical evaluation of the severity of SCI is critical to creating a suitable treatment plan. Utilizing rodent models is the first step towards creating metabolomic profiles with four varying degrees of SCI. The degrees of SCI included a control (sham), light injury, moderate injury, and heavy injury groups. Through surgical methods, CSF, plasma, and spinal cord were used as samples in sub-acute SCI rodents. LC–MS metabolomic profiling revealed that 130,104 and 128 metabolites were altered in the samples taken from the rodent population [58].

Furthermore, four differential metabolites, uric acid, phosphorylcholine, pyridoxine, and guanidinoacetic acid, were identified within the varying degrees of SCI. Specifically in regard to uric acid, this increase can be attributed to tissue and cellular damage in the forms of purine breakdown and oxidative stress. However, the increase in uric acid increase can also act as a protector for prevention of further secondary spinal cord injury damage [59]. Further research should be conducted to observe if consistent findings are found using metabolomic profiling. Thus, if results are consistent, the omics method can be used in addition to the other methods mentioned to identify SCI severity, leading to better-suited treatment plans [58].

With metabolomic profiling becoming more widely understood, combined omics approaches offer the potential for significant advancements, such as a combined transcriptomic and metabolomic study utilizing rodent models. Substantial changes in metabolites and genes displayed the continued success of omics approaches in analyzing SCI. SCI treatment plans are key interest points for future research, and the potential for combined omics approaches allows for greater information to be gathered. As seen in Table 1, the various different omics appraoches and studies utilized display specific and critical information. Thus, creating SCI treatment plans tailored to the individual continues to be further researched and developed for potential future clinical application [60].

## 5. Emerging Therapeutic Strategies Targeting Molecular Pathways

### 5.1. Channel Blockers

One way to prevent glutamate activation that leads to excitotoxicity is to use Na^+^ channel blockers, which inhibit cell depolarization and prevent glutamate release from neurons [15]. These channel blockers are used to prevent ion imbalances that lead to excitotoxicity damage. Common sodium channel blockers include tetrodotoxin, riluzole, mexiletine, lamotrigine, and phenytoin. In a study specifically using riluzole and phenytoin in a rodent population, researchers tested the neuroprotective capabilities. Researchers injected Wistar rats intraperitoneally with riluzole (5 mg/kg), phenytoin (30 mg/kg), CNS5546A, novel Na^+^ channel blocker, or vehicle (2-HP3CD; 5 mg/kg) 15 min after induction of compressive SCI at C7-T1. Functional recovery of the hindlimbs in regard to function and strength was assessed weekly post injury for 6 weeks. The overall results were positive, specifically with riluzole, thus furthering the use of Na^+^ channel blockers in SCI treatments. The preservation of residual tissue and descending axon functionality were key indicators of this treatment success [58]. While this study was conducted in a mouse model, it shows great potential for human translation. Ion channel blockers are currently commonly used for neuropathic pain treatment in humans, including mexiletine, lamotrigine, gabapentin, and pregabalin for SCI [61]. Gabapentin and pregabalin reduce Ca^2+^ release. Adverse effects of sodium channel blockers include arrhythmias, hypotension, and seizures [62].

### 5.2. Antioxidants

Antioxidants are aimed at mitigating the damage from or preventing oxidative stress. Omega-3 fatty acids and docosahexaenoic acid have anti-inflammatory and antioxidant properties that inhibit the production of reactive oxygen species and lipid peroxidation [15]. Commonly, glucocorticoids like dexamethasone and methylprednisolone are treatments that inhibit ROS formation. However, as shown in some studies, methylprednisolone does not appear to have a significant impact on recovery, including motor or sensory function [63,64,65]. Therefore, recent guidelines proposed by the American Association of Neurological Surgeons and the Congress of Neurological Surgeons in 2013 recommend limited administration of these glucocorticoids [15]. The limitation was partly proposed due to insufficient evidence supporting clinical benefit and the risk of side effects, including increased risk of infection and gastrointestinal bleeding [66,67,68]. Additionally, of note, glucocorticoids are complicated and can sometimes be pro-oxidants, especially in high concentrations [69,70,71]. Further, Ca^2+^ channel blockers, including nimodipine, mibefradil, and trimethadione, can prevent an influx of calcium that triggers the release of ROS. Recently, several antioxidants have been found to be promising in regulating oxidative stress [15]. These antioxidants include U-83836E and tempol, inhibiting lipid peroxidation, and melatonin, a free radical scavenger [72,73,74]. In broad terms, antioxidants act as neuroprotective agents that regulate ROS.

### 5.3. Mitochondrial Repair and Transplantation

Previous animal model studies have demonstrated mitochondrial transplantation as a promising SCI treatment [42]. Damaged mitochondria can restore their integrity by fusing with healthy mitochondria to exchange DNA, protein, and metabolites, or they can be degraded through mitophagy. Mitochondrial fission separates healthy and damaged mitochondrial components. Mitochondrial transplantation has applications in other fields such as post-ischemia reperfusion and Parkinson’s disease [75,76]. While at its early stages of testing for SCI treatment, mitochondrial repair has shown some promising results in numerous animal studies [76,77,78]. Transplantation of exogenous mitochondria has been shown to reduce intracellular ROS and improve calcium buffering capacity, essential mitigators to reduce SCI chronic burden [42]. As seen in Table 2, the various mechanisms describe in emerging therapuetic strategies seek to tackle different aspects of spinal cord injuries as current treatment methods are expanded upon and further developed.

## 6. Discussion and Future Directions

While the most common cause of SCI remains motor vehicle accidents and falls, there are other non-traumatic causes of note. Cervical myelopathy is characterized by hand clumsiness, neck pain, arm numbness or tingling, and difficulty walking. It is often related to aging and caused by cervical spinal cord compression in the neck [78,79,80]. It is a leading cause of spinal cord dysfunction in older adults [75]. If left untreated, cervical myelopathy may lead to paralysis [81]. Additionally, spinal cord strokes, when blood flow is obstructed to the spinal cord, can lead to paralysis [82,83]. Ischemia, restricted blood flow, to the spinal cord can lead to neuronal cell death and activate the secondary injury phase that contributes to disability. Lastly, there are several case reports of paralysis as a complication following spinal cord surgery [84,85]. This potential complication is an important risk factor to discuss with patients.

While there is currently encouraging animal model data that supports the use of mitochondrial transplantation for SCI treatment, more research remains to be conducted to ensure successful translation into a clinical setting [42]. Translational work includes determining time and dosage effects. Furthermore, the molecular mechanisms behind mitochondrial transplantation remain to be solidified and require further investigation to understand the underlying cellular response.

Multiple destructive processes, including excitotoxicity, oxidative stress, mitochondrial dysfunction, and inflammation, can describe the pathophysiology of SCI. Furthermore, each process contributes to and exacerbates neuronal and glial cell death [17,18,19,38,39]. As a result, recovery is impaired, and, overall, chronic pain further sets in. Firstly, excitotoxicity, attributed to glutamate overload and dysregulated calcium influx via ligand-gated NMDA receptors, leads to cell death [39]. Additionally, oxidative stresses, which are connected to reactive oxygen species, lead to further destabilization and diminishing cellular integrity [42,43,44]. There is damage to the lipids, proteins, and DNA. Together, these two issues lead to impairment in mitochondrial function, critical to cellular energy [44].

The post traumatic inflammatory response can lead to greater damage if unregulated, causing increased pain. The infiltration of neutrophils, macrophages, and microglia leads to the release of pro-inflammatory cytokines. These pro-inflammatory cytokines include TNF-α, IL-1β, and IL-6 [19,36]. If the inflammation persists over an extended period, it leads to increased tissue damage, inhibited recovery, and glial scars [38].

With these factors in mind, the omics technologies are beginning to show promising results in combating and contributing to SCI treatment plans. This also further builds upon precision medicine approaches and the layers of complexity seen in some SCI cases. Genomics, proteomics, and metabolomics offer different information to help create a cohesive SCI treatment plan. Genomic technologies include sequencing and GWAS to help better identify specific risk factors regarding genetics [48]. Furthermore, a subsection of genomics, transcriptomics, utilizes RNA-sequencing, which helps determine gene expression alterations [48,49]. Tracking these gene expression changes is crucial for defining and identifying potential adverse outcomes when using an SCI treatment plan. Transcriptomics has given valuable, time-sensitive information following the idea of tracking gene expression and further contributes to the progression of precision medicine. Notably, IL-10 has also become a cytokine of interest with its anti-inflammatory and neuroprotective capabilities [38].

Consequently, proteomics in acute SCI has allowed for greater information regarding protein expression to be found. Specifically, greater analysis of protein expression patterns can be seen, with the potential for biomarkers to be found. Based on findings, these biomarkers can then be attributed to injury severity and functional outcomes. Our review analyzed CSF and serum samples in animal and human studies, which displayed significant protein differences between control and SCI groups [57]. The implications of proteomics in cases of acute SCI are of great interest, especially with the potential for biomarkers providing great information and aid in curated, tailored SCI treatment plans.

Lastly, metabolomics also contributes to furthering SCI treatment plans. Metabolomics allows for greater information in biochemical changes involving the severity of SCI present [49]. Our review highlights metabolites such as uric acid, pyridoxine, and phosphorylcholine [58]. These metabolites serve as biomarkers, which provide great context in evaluating the severity of SCI in the patient. Metabolomic profiles can be great tools for assessing treatment plans in the early stages and analyzing shifts in metabolic profiles as the SCI changes over time [60].

There have also been some studies examining the potential for combinatory omics approaches [13]. Further research analyzing all three approaches of genomics, proteomics, and metabolomics should be conducted to test if these options provide greater benefit than each method individually. All the approaches together would yield the greatest SCI treatment plan as each provides different information. The study used in our review highlights an approach containing the genomic subsection of transcriptomic and metabolomic, with results proving beneficial [13].

The implications of the findings discussed in this review are significant regarding therapeutic options. Honing in ion imbalance in sodium and calcium channel blockers has led to promising models of excitotoxicity mitigation [15]. Furthermore, antioxidants (melatonin, tempol, and omega-3 fatty acids) have been shown to reduce oxidative stress and maintain mitochondrial function [72,73,74]. Additionally, glucocorticosteroids like methylprednisolone are also in limited studies.

An exciting area of growth in SCI treatment involves mitochondrial therapy. This therapeutic method is related to approaches involving mitochondrial transplantation, which has been shown to have the potential of restoring cellular energy balance, decreasing ROS production, and improving overall outcomes [77,78]. The studies found are only animal models at this time; however, there have been promising results in these initial studies [42,74,75,76,77,78]. Some challenges that remain include dosing, delivery mechanisms, and safety.

While promising results have been seen through the omics approaches, significant hurdles remain. There are particular challenges in translating the findings from animal to human studies when analyzing SCI regarding the injury’s severity, location, and timing [86]. Additional clinical trials will be needed to validate the safety and efficacy of omics therapies and mitochondrial-based therapeutics. Developing methods for scaling and reducing costs for the tools to utilize omics approaches is an additional challenge.

Medicine has continued shifting towards artificial intelligence (AI) integration, and omics is no different. AI and machine learning regarding omics approaches could potentially revolutionize biomarker discovery and identification, which only speeds up potential mainstream clinical application of these methods [87]. AI and machine learning would aid significantly in identifying SCI patterns with increased information, thus leading to the development of even greater treatment plans much faster.

Although there are many innovations with great promise, many therapeutics have not yet translated into clinical outcome improvements. For one, SCI pathophysiology is complex and involves many different cell types and mechanisms. One single approach is often not enough [88]. Similarly, translation from animal models to clinical settings in humans is difficult as their anatomy and conditions are not exactly comparable, and there is a higher degree of diversity in humans [85]. For example, lesion sizes and location may be different, calling for the need for a diverse animal cohort [88]. Furthermore, there may be challenges with dosing and delivery in translational studies. Additionally, timing is critical to treating SCI, as found in numerous animal models [89,90]. In a clinical setting, patients may not be able to immediately seek medical attention for various reasons, including systemic barriers to accessing healthcare, but rather when their condition is chronic. Animal studies are conducted under ideal, acute conditions [91]. Timing is an additional factor that needs to be considered in SCI animal models to fully encompass individual diversity.

## 7. Conclusions

Traumatic SCI is a devastating condition for the patient and all caregivers involved in treatment. Additionally, it carries a significant financial burden because life care planning often involves restructuring of the home to adapt and loss of wages for the patient and loved ones involved in daily management. Our review discussed the importance of understanding SCI pathophysiology, molecular mechanisms, and therapeutic targets. Beginning with a discussion of the primary and secondary injury phases, an introduction to the progression of SCI is necessary to understand the complexity of tailored treatment plans. The secondary injury phase, in particular, is of greater significance regarding deploying omics approaches. This information is important to forecast the direction of translational medicine towards possible treatment options.

Overall, SCI treatment plan research is constantly growing and evolving. Our review covers foundational knowledge of the phases of injury and cellular mechanisms, omics technologies, and emerging therapies and looks towards the future with AI and machine learning integration. Omics approaches are helping drive this new era of precision medicine, and understanding how it relates to a devastating condition such as SCI is paramount. By deepening the current understanding of SCI at the molecular level, creating tailored SCI treatment plans is closer to reality than ever.

## Figures and Tables

**Table 1:** Omics approaches and key information gained from each method.

Omics Approach	Key Information	References
Genomics	DNA Mutations ○ Insertions ○ Deletions RNA Sequencing (Transcriptomics) Epigenetics	Micheel C.M. et al. [47] Gasperskaja et al. [48] Hasin et al. [49] Zhang et al. [53] Mun et al. [54]
Proteomics	Protein Structural stability Post-translational modifications	Gasperskaja et al. [48] Liu et al. [56] Skinnider et al. [57]
Metabolomics	Metabolites Environmental and physiological changes Metabolic pathway activity	Hasin et al. [49] Yang et al. [58] Zeng et al. [60]

**Table 2:** Therapeutic strategies for SCI injury mechanisms.

Mechanism	Therapeutic Strategy	Examples	Key Effects
Ion Imbalance	Na^+^ Channel Blockers	Tetrodotoxin, riluzole, mexiletine, phenytoin	Inhibit neuronal depolarization, which prevent glutamate release and excitotoxicity
Oxidative Stress	Antioxidants	U-83836E, tempol, melatonin	Inhibit ROS production and lipid peroxidation; scavenge free radicals
	Glucocorticoids	Dexamethasone, methylprednisolone	Inhibit ROS; limited use due to side effects
	Ca^2+^ Channel Blockers	Nimodipine, mibefradil, trimethadione	Reduce calcium influx and prevent ROS-triggering pathways
Mitochondrial Repair and Transplantation	Mitochondrial Transplantation	Exogenous mitochondrial injection	Enhances calcium buffering, reduces ROS, restores cellular function
	Endogenous Repair	Mitochondrial fusion, fission, mitophagy	Promotes removal or repair of damaged mitochondria

## References

[1] National Institute of Neurological Disorders and Stroke (2025). Spinal Cord Injury.

[2] Khorasanizadeh M., Yousefifard M., Eskian M., Lu Y., Chalangari M., Harrop J.S., Jazayeri S.B., Seyedpour S., Khodaei B., Hosseini M. (2019). Neurological recovery following traumatic spinal cord injury: A systematic review and meta-analysis. J. Neurosurg. Spine.

[3] World Health Organization (2024). Spinal Cord Injury.

[4] GBD Spinal Cord Injuries Collaborators (2023). Global, regional, and national burden of spinal cord injury, 1990–2019: A systematic analysis for the Global Burden of Disease Study 2019. Lancet Neurol..

[5] (2017). pinal Cord Injury Facts and Figures at a Glance.

[6] Greitemeyer T., Kastenmüller A., Fischer P. (2012). Romantic motives and risk-taking: An evolutionary approach. J. Risk Res..

[7] Stein D.G., Hoffman S.W. (2003). Estrogen and progesterone as neuroprotective agents in the treatment of acute brain injuries. Pediatr. Rehabil..

[8] Bramlett H.M., Dietrich W.D. (2004). Neuropathological Protection after Traumatic Brain Injury in Intact Female Rats Versus Males or Ovariectomized Females. J. Neurotrauma.

[9] Lima R., Monteiro A., Salgado A.J., Monteiro S., Silva N.A. (2022). Pathophysiology and Therapeutic Approaches for Spinal Cord Injury. Int. J. Mol. Sci..

[10] Rowland J.W., Hawryluk G.W., Kwon B., Fehlings M.G. (2008). Current status of acute spinal cord injury pathophysiology and emerging therapies: Promise on the horizon. Neurosurg. Focus.

[11] Wang L., Qu J., Harari O., Boddey J.A., Wang Z., Linna-Kuosmanen S. (2024). The impact of multi-omics in medicine. Cell Rep. Med..

[12] Peng R., Zhang L., Xie Y., Guo S., Cao X., Yang M. (2024). Spatial multi-omics analysis of the microenvironment in traumatic spinal cord injury: A narrative review. Front. Immunol..

[13] Mackiewicz-Milewska M., Newland P. (2016). Spinal Cord Injury (SCI) 2016 Facts and Figures at a Glance. J. Spinal Cord. Med..

[14] Anjum A., Yazid M.D., Daud M.F., Idris J., Ng A.M.H., Naicker A.S., Ismail O.H.R., Kumar R.K.A., Lokanathan Y. (2020). Spinal Cord Injury: Pathophysiology, Multimolecular Interactions, and Underlying Recovery Mechanisms. Int. J. Mol. Sci..

[15] Couillard-Despres S., Bieler L., Vogl M., Weidner N., Rupp R., Tansey K. (2017). Pathophysiology of Traumatic Spinal Cord Injury. Neurological Aspects of Spinal Cord Injury.

[16] Park E., Velumian A.A., Fehlings M.G. (2004). The role of excitotoxicity in secondary mechanisms of spinal cord injury: A review with an emphasis on the implications for white matter degeneration. J. Neurotrauma.

[17] Hall E.D., Wang J.A., Bosken J.M., Singh I.N. (2016). Lipid peroxidation in brain or spinal cord mitochondria after injury. J. Bioenerg. Biomembr..

[18] Hellenbrand D.J., Quinn C.M., Piper Z.J., Morehouse C.N., Fixel J.A., Hanna A.S. (2021). Inflammation after spinal cord injury: A review of the critical timeline of signaling cues and cellular infiltration. J. Neuroinflamm..

[19] Yang L., Shi F., Cao F., Wang L., She J., He B., Xu X., Kong L., Cai B. (2025). Neutrophils in Tissue Injury and Repair: Molecular Mechanisms and Therapeutic Targets. MedComm.

[20] Kulkarni O.P., Lichtnekert J., Anders H.-J., Mulay S.R. (2016). The Immune System in Tissue Environments Regaining Homeostasis after Injury: Is “Inflammation” Always Inflammation?. Mediat. Inflamm..

[21] Greenwood S., Connolly C.N. (2007). Dendritic and Mitochondrial Changes During Glutamate Excitotoxicity. Neuropharmacology.

[22] Kritis A., Stamoula E.G., Paniskaki K.A., Vavilis T.D. (2015). Researching Glutamate—Induced Cytotoxicity in Different Cell Lines: A Comparative/Collective Analysis/Study. Front. Cell. Neurosci..

[23] Ayala A., Muñoz M.F., Argüelles S. (2014). Lipid peroxidation: Production, metabolism, and signaling mechanisms of malondialdehyde and 4-hydroxy-2-nonenal. Oxidative Med. Cell. Longev..

[24] Ademowo O.S., Dias H.K.I., Burton D.G.A., Griffiths H.R. (2017). Lipid (per) oxidation in mitochondria: An emerging target in the ageing process?. Biogerontology.

[25] Szczepanowska J., Malinska D., Wieckowski M.R., Duszynski J. (2012). Effect of mtDNA Point Mutations on Cellular Bioenergetics. Biochim. Biophys. Acta (BBA) Bioenerg..

[26] Vakifahmetoglu-Norberg H., Ouchida A.T., Norberg E. (2017). The Role of Mitochondria in Metabolism and Cell Death. Biochem. Biophys. Res. Commun..

[27] Cantó-Santos J., Grau-Junyent J.M., Garrabou G. (2020). The Impact of Mitochondrial Deficiencies in Neuromuscular Diseases. Antioxidants.

[28] Shi Z., Yuan S., Shi L., Li J., Ning G., Kong X., Feng S. (2021). Programmed cell death in spinal cord injury pathogenesis and therapy. Cell Prolif..

[29] Hausmann O. (2003). Post-traumatic inflammation following spinal cord injury. Spinal Cord..

[30] Shuman S.L., Bresnahan J.C., Beattie M.S. (1997). Apoptosis of microglia and oligodendrocytes after spinal cord contusion in rats. J. Neurosci. Res..

[31] Liu X.Z., Xu X.M., Hu R., Du C., Zhang S.X., McDonald J.W., Dong H.X., Wu Y.J., Fan G.S., Jacquin M.F. (1997). Neuronal and glial apoptosis after traumatic spinal cord injury. J. Neurosci. Off. J. Soc. Neurosci..

[32] MMoura M.M., Monteiro A., Salgado A.J., Silva N.A., Monteiro S. (2024). Disrupted autonomic pathways in spinal cord injury: Implications for the immune regulation. Neurobiol. Dis..

[33] Elmalky M.I., Alvarez-Bolado G., Younsi A., Skutella T. (2024). Axonal Regeneration after Spinal Cord Injury: Molecular Mechanisms, Regulatory Pathways, and Novel Strategies. Biology.

[34] Nakamura M., Okada S., Toyama Y., Okano H. (2005). Role of IL-6 in spinal cord injury in a mouse model. Clin. Rev. Allerg. Immunol..

[35] Patel M., Wahezi S., Mavrocordatos P., Abd-Elsayed A. (2025). The Effects and Mechanisms of Phytochemicals on Pain Management and Analgesic. Nutrients.

[36] Ji R.-R., Nackley A., Huh Y., Terrando N., Maixner W. (2018). Neuroinflammation and Central Sensitization in Chronic and Widespread Pain. Anesthesiology.

[37] Patilas C., Varsamos I., Galanis A., Vavourakis M., Zachariou D., Marougklianis V., Kolovos I., Tsalimas G., Karampinas P., Kaspiris A. (2024). The Role of Interleukin-10 in the Pathogenesis and Treatment of a Spinal Cord Injury. Diagnostics.

[38] Jewett B.E., Thapa B. (2025). Physiology, NMDA Receptor. StatPearls.

[39] Wang Q., Wang X., Shang Z., Zhao L. (2024). Mechanism and prospects of mitochondrial transplantation for spinal cord injury treatment. Stem Cell Res. Ther..

[40] Kim K., Mishina M., Kokubo R., Nakajima T., Morimoto D., Isu T., Kobayashi S., Teramoto A. (2013). Ketamine for acute neuropathic pain in patients with spinal cord injury. J. Clin. Neurosci. Off. J. Neurosurg. Soc. Australas..

[41] Tang S., Yu J., Li J., Sun J. (2015). Neuroprotective effect of ketamine on acute spinal cord injury in rats. Genet. Mol. Res. GMR.

[42] Yu M., Wang Z., Wang D., Aierxi M., Ma Z., Wang Y. (2023). Oxidative stress following spinal cord injury: From molecular mechanisms to therapeutic targets. J. Neurosci. Res..

[43] Juan C.A., de la Lastra J.M.P., Plou F.J., Pérez-Lebeña E. (2021). The Chemistry of Reactive Oxygen Species (ROS) Revisited: Outlining Their Role in Biological Macromolecules (DNA, Lipids and Proteins) and Induced Pathologies. Int. J. Mol. Sci..

[44] Bradbury E.J., Burnside E.R. (2019). Moving beyond the glial scar for spinal cord repair. Nat. Commun..

[45] Li S., Ohtake Y., Smith G. (2016). Reactive astrocyte scar and axon regeneration: Suppressor or facilitator?. Neural Regen. Res..

[46] Micheel C.M., Nass S.J., Omenn G.S., Committee on the Review of Omics-Based Tests for Predicting Patient Outcomes in Clinical Trials, Board on Health Care Services, Board on Health Sciences Policy, Institute of Medicine (2012). 2. Omics-Based Clinical Discovery: Science, Technology, and Applications. Evolution of Translational Omics: Lessons Learned and the Path Forward.

[47] Gasperskaja E., Kučinskas V. (2017). The most common technologies and tools for functional genome analysis. Acta Medica Litu..

[48] Hasin Y., Seldin M., Lusis A. (2017). Multi-omics approaches to disease. Genome Biol..

[49] Alberts B., Johnson A., Lewis J., Raff M., Roberts K., Walter P. (2002). From DNA to RNA. Molecular Biology of the Cell.

[50] Williamson A.K., Zhu Z., Yuan Z.-M. (2018). Epigenetic mechanisms behind cellular sensitivity to DNA damage. Cell Stress.

[51] Lambert S., Jolma A., Campitelli L.F., Das P.K., Yin Y., Albu M., Chen X., Taipale J., Hughes T.R., Weirauch M.T. (2018). The Human Transcription Factors. Cell.

[52] Chang P.-Y., Zhang B.-Y., Zhu Q.-S., Zhu Y.-H., Saijilafu (2020). Decoding epigenetic codes: New frontiers in exploring recovery from spinal cord injury. Neural Regen. Res..

[53] Mun S., Han K., Hyun J.K. (2022). The Time Sequence of Gene Expression Changes after Spinal Cord Injury. Cells.

[54] Liu R., Zhao W., Zhao Q., Liu S.-J., Liu J., He M., Xu Y., Wang W., Liu W., Xia Q.-J. (2014). Endoplasmic reticulum protein 29 protects cortical neurons from apoptosis and promoting corticospinal tract regeneration to improve neural behavior via caspase and Erk signal in rats with spinal cord transection. Mol. Neurobiol..

[55] https://pmc.ncbi.nlm.nih.gov/articles/PMC11811828/.

[56] Skinnider M.A., Rogalski J., Tigchelaar S., Manouchehri N., Prudova A., Jackson A.M., Nielsen K., Jeong J., Chaudhary S., Shortt K. (2021). Proteomic Portraits Reveal Evolutionarily Conserved and Divergent Responses to Spinal Cord Injury. Mol. Cell. Proteom. MCP.

[57] Yang H., Zhang P., Xie M., Luo J., Zhang J., Zhang G., Wang Y., Lin H., Ji Z. (2022). Parallel Metabolomic Profiling of Cerebrospinal Fluid, Plasma, and Spinal Cord to Identify Biomarkers for Spinal Cord Injury. J. Mol. Neurosci..

[58] Zeng Z., Li M., Jiang Z., Lan Y., Chen L., Chen Y., Li H., Hui J., Zhang L., Hu X. (2022). Integrated transcriptomic and metabolomic profiling reveals dysregulation of purine metabolism during the acute phase of spinal cord injury in rats. Front. Neurosci..

[59] Scott G.S., Cuzzocrea S., Genovese T., Koprowski H., Hooper D.C. (2005). Uric acid protects against secondary damage after spinal cord injury. Proc. Natl. Acad. Sci. USA.

[60] Schwartz G., Fehlings M.G. (2001). Evaluation of the neuroprotective effects of sodium channel blockers after spinal cord injury: Improved behavioral and neuroanatomical recovery with riluzole. J. Neurosurg..

[61] Chen L., Mao J. (2013). Update on Neuropathic Pain Treatment: Ion Channel Blockers and Gabapentinoids. Curr. Pain. Headache Rep..

[62] Dokken K., Chen R.J., Fairley P. (2025). Sodium Channel Blocker Toxicity. StatPearls.

[63] Evaniew N., Noonan V.K., Fallah N., Kwon B.K., Rivers C.S., Ahn H., Bailey C.S., Christie S.D., Fourney D.R., Hurlbert R.J. (2015). Methylprednisolone for the Treatment of Patients with Acute Spinal Cord Injuries: A Propensity Score-Matched Cohort Study from a Canadian Multi-Center Spinal Cord Injury Registry. J. Neurotrauma.

[64] Canseco J., Karamian B.A., Bowles D.R., Markowitz M.P., DiMaria S.L., Semenza N.C., Leibensperger M.R., Smith M.L., Vaccaro A.R. (2021). Updated Review: The Steroid Controversy for Management of Spinal Cord Injury. World Neurosurg..

[65] Miękisiak G., Łątka D., Jarmużek P., Załuski R., Urbański W., Janusz W. (2019). Steroids in Acute Spinal Cord Injury: All But Gone Within 5 Years. World Neurosurg..

[66] Narum S., Westergren T., Klemp M. (2014). Corticosteroids and risk of gastrointestinal bleeding: A systematic review and meta-analysis. BMJ Open.

[67] Youssef J., Novosad S.A., Winthrop K.L. (2016). Infection Risk and Safety of Corticosteroid Use. Rheum. Dis. Clin. N. Am..

[68] Mustafa A.G., Singh I.N., Wang J., Carrico K.M., Hall E.D. (2010). Mitochondrial protection after traumatic brain injury by scavenging lipid peroxyl radicals. J. Neurochem..

[69] McIntosh L.J., Sapolsky R.M. (1996). Glucocorticoids increase the accumulation of reactive oxygen species and enhance adriamycin-induced toxicity in neuronal culture. Exp. Neurol..

[70] Iuchi T., Akaike M., Mitsui T., Ohshima Y., Shintani Y., Azuma H., Matsumoto T. (2003). Glucocorticoid excess induces superoxide production in vascular endothelial cells and elicits vascular endothelial dysfunction. Circ. Res..

[71] Sato H., Takahashi T., Sumitani K., Takatsu H., Urano S. (2010). Glucocorticoid Generates ROS to Induce Oxidative Injury in the Hippocampus, Leading to Impairment of Cognitive Function of Rats. J. Clin. Biochem. Nutr..

[72] Vaishnav R.A., Singh I.N., Miller D.M., Hall E.D. (2010). Lipid peroxidation-derived reactive aldehydes directly and differentially impair spinal cord and brain mitochondrial function. J. Neurotrauma.

[73] Samantaray S., Das A., Thakore N.P., Matzelle D.D., Reiter R.J., Ray S.K., Banik N.L. (2009). Therapeutic potential of melatonin in traumatic central nervous system injury. J. Pineal Res..

[74] McCully J.D., Cowan D.B., Pacak C.A., Toumpoulis I.K., Dayalan H., Levitsky S. (2009). Injection of isolated mitochondria during early reperfusion for cardioprotection. Am. J. Physiol. Heart Circ. Physiol..

[75] Eo H., Yu S.-H., Choi Y., Kim Y., Kang Y.C., Lee H., Kim J.H., Han K., Lee H.K., Chang M.-Y. (2024). Mitochondrial transplantation exhibits neuroprotective effects and improves behavioral deficits in an animal model of Parkinson’s disease. Neurother. J. Am. Soc. Exp. Neurother..

[76] Gollihue J.L., Rabchevsky A.G. (2017). Prospects for therapeutic mitochondrial transplantation. Mitochondrion.

[77] Gollihue J.L., Patel S.P., Eldahan K.C., Cox D.H., Donahue R.R., Taylor B.K., Sullivan P.G., Rabchevsky A.G. (2018). Effects of Mitochondrial Transplantation on Bioenergetics, Cellular Incorporation, and Functional Recovery after Spinal Cord Injury. J. Neurotrauma.

[78] Donnally C.J., Hanna A., Odom C.K. (2025). Cervical Myelopathy. StatPearls.

[79] Al-Ryalat N.T., AlRyalat S.A.S., Mahafza W.S., Samara O.A., Ryalat A.T., Al-Hadidy A.M. (2017). Myelopathy associated with age-related cervical disc herniation: A retrospective review of magnetic resonance images. Ann. Saudi Med..

[80] Milligan J., Ryan K., Fehlings M., Bauman C. (2019). Degenerative cervical myelopathy: Diagnosis and management in primary care. Can. Fam. Physician.

[81] Patel S., Naidoo K., Thomas P. (2014). Spinal cord infarction: A rare cause of paraplegia. BMJ Case Rep..

[82] Lee S.-H., Kim S.B., Choi S.G., Lim Y.J. (2008). Paraplegia due to Spinal Cord Infarction After Lifting Heavy Objects. J. Korean Neurosurg. Soc..

[83] Hashimoto M., Mochizuki M., Aiba A., Okawa A., Hayashi K., Sakuma T., Takahashi H., Koda M., Takahashi K. (2010). C5 palsy following anterior decompression and spinal fusion for cervical degenerative diseases. Eur. Spine J. Off. Publ. Eur. Spine Soc. Eur. Spinal Deform. Soc. Eur. Sect. Cerv. Spine Res. Soc..

[84] Fang M., Zhou J., Zeng Y., Huang S., Song Y. (2022). Conversion Paralysis After Cervical Surgery: A Case Report and Literature Review. Front. Surg..

[85] Stewart A.N., Gensel J.C., Jones L., Fouad K. (2023). Challenges in Translating Regenerative Therapies for Spinal Cord Injury. Top. Spinal Cord. Inj. Rehabil..

[86] Gao F., Huang K., Xing Y. (2022). Artificial Intelligence in Omics. Genom. Proteom. Bioinform..

[87] Tian T., Zhang S., Yang M. (2023). Recent progress and challenges in the treatment of spinal cord injury. Protein Cell.

[88] Akhtar A.Z., Pippin J.J., Sandusky C.B. (2008). Animal models in spinal cord injury: A review. Rev. Neurosci..

[89] Ahuja C.S., Badhiwala J.H., Fehlings M.G. (2020). “Time is spine”: The importance of early intervention for traumatic spinal cord injury. Spinal Cord..

[90] Li M.-Q., Wang Q.H., Dong C.M., Qi L.J. (2025). Spinal Cord Injury Models: Advantages and Disadvantages in the View of Pathophysiology and Clinical Significance. Biochem. Biophys. Rep..

[91] Gregory N.S., Harris A.L., Robinson C.R., Dougherty P.M., Fuchs P.N., Sluka K.A. (2013). An overview of animal models of pain: Disease models and outcome measures. J. Pain..

